# Impacts of Waste Rubber Products on the Structure and Properties of Modified Asphalt Binder: Part I—Crumb Rubber

**DOI:** 10.3390/ma17194685

**Published:** 2024-09-24

**Authors:** Svetlana Obukhova, Angelina Budkina, Evgeniy Korolev, Vitaliy Gladkikh

**Affiliations:** 1 Department of Urban Planning, Institute of Architecture and Urban Planning, National Research Moscow State University of Civil Engineering, Moscow 129337, Russia; angelina-line@yandex.ru; 2 Scientific and Educational Center “Nanomaterials and Nanotechnologies”, National Research Moscow State University of Civil Engineering, Moscow 129337, Russia; korolev@nocnt.ru; 3 Research Center «MGSU Stroy-Test», National Research Moscow State University of Civil Engineering, Moscow 129337, Russia; gladkich_87@mail.ru

**Keywords:** crumb rubber, crumb-rubber-modified bitumen, structure, compatibility, stability, solubility, thermodynamic compatibility parameters, sustainable

## Abstract

The issue of forming a reliable and sustainable structure of crumb-rubber-modified binder is an important scientific and technical task. The quality of this task will increase the technical and economic efficiencies of road construction materials. This work is dedicated to developing a scientifically justified method of directed thermomechanical devulcanization, which ensures the solubility of the crumb rubber in the complex structure of a polydisperse composite material, preventing the formation of aggregates consisting of unsaturated crumb rubber particles, whose elastic aftereffect causes intensive cracking, especially during low-temperature road operations. The novelty in the first part of this article is due to the fact that, for the first time, the quantitative ratio of the polymer component in the crumb rubber was experimentally determined. The ratio of the polymer component to the total content of the other rubber components in the crumb rubber (CR) was determined to be, on average, 93.3 ± 1.8%. The stabilities of the compositions of crumb rubber from different batches were experimentally studied. The nature of the polymer component in the crumb rubber was determined. A hypothesis was formulated to obtain a thermodynamically stable and sustainable binder modified with crumb rubber. To evaluate the compatibility of hydrocarbon plasticizers with the studied CR samples, the following semi-empirical and thermodynamic compatibility parameters were calculated: Hildebrand solubility parameters based on evaporation energy and surface tension, Barstein’s compatibility parameter |X|, Traxler coefficient, and the mass ratio of paraffin naphthene:asphaltenes. It was shown that for the substances under study, it is advisable to justify the choice of plasticizer based on chemical compatibility criteria. It was established that a supramolecular plasticization mechanism occurs in the “hydrocarbon plasticizer–crumb rubber” systems under consideration. In the development of the crumb-rubber-modified binder, it was found that the use of activated crumb rubber (ACR) from large tires does not ensure the achievement of a stable and resilient structure of the crumb-rubber-modified bitumen.

## 1. Introduction

**Figure 1:**
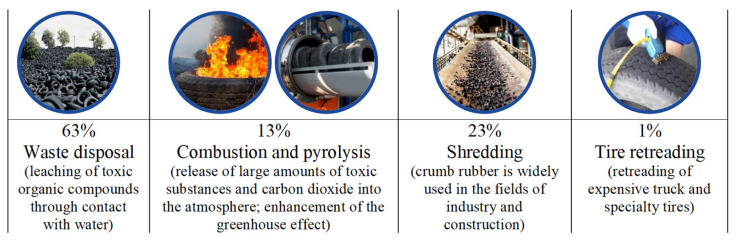
Ways to recycle used tires.

Road networks are a crucial element of any nation’s economy. Their efficient functioning and sustainable development are essential prerequisites for a transition toward economic growth, ensuring the integrity and national security of a country and improving the standards and quality of people’s lives. This necessitates the creation of reliable and safe roads for both people and the environment. At the same time, the construction of new roads and maintenance of existing ones require unlimited access to resources, including bitumen. This requirement imposes economic and environmental constraints on the road construction industry. The need for affordable and high-quality resources while also respecting environmental standards requires an evaluation of the products used throughout their life cycle. An additional factor is the annual increase in the automotive and consumer markets, which complicates the situation in terms of environmental safety. Global reserves of rubber product waste collectively represent a significant volume, reaching around 80 million tons, with a constant annual increase of at least 10% in total reserves [1,2]. Waste from used tires falls under the IV hazard class and takes 12 to 140 years to decompose in the environment. Presently, there are several ways to recycle used tires (see Figure 1) [2].

Based on the provided data (Figure 1), crumb rubber grinding appears to be the most attractive method of disposal to minimize the environmental damage. However, it is important to note that as the scale of this method’s implementation increases, significant reserves of unused ground crumb rubber (CR) are generated. This underscores the relevance of research into the potential for effectively utilizing crumb rubber, including as a modifying component in bitumen binders [3].

The quality of the bitumen directly depends on the state of its structure. According to the “classical colloidal theory” of bitumen’s structure, proposed by Nellensteyn, in 1923 [4], its structure consists of lyophobic asphaltenes surrounded by lyophilic resins, which form an adsorption–solvation shell that prevents the aggregation of asphaltenes. These colloidal formations are micelles distributed in an oily medium. The micelle core can be a particle composed of carbon surrounded by adsorbed asphaltenes. Later, Traxler [5] developed this theory, establishing that the most polar, aromatic, and high-molecular-weight components are located in the micelle core, and as the distance from the core increases, the polarity and molecular weight of the components decrease. This theory, known as “colloidal theory”, is based on the Tyndall cone effect (i.e., light scattering) in bitumen solutions in benzene and the ability of their dispersed components to undergo Brownian motion. Depending on the concentration and liposomal properties of the asphaltenes, bitumen mycelia may interact with each other through thin layers of the dispersion medium (oil), either forming a coagulated structure or remaining stabilized as a solution. As a result, the bitumen binder acquires the properties of a gel, sol, or an intermediate colloidal structure of the sol-gel type [6]. However, it should be noted that this theory does not explain the thermodynamic stability of bitumen and even suggests the heterogeneity and instability of the system.

The development of the colloidal theory of petroleum-based dispersive systems (“modern colloidal systems”) was furthered in the works of Syunyaev [7]. He considered the dispersive phase of bitumen as a system composed of complex structural units (CSUs), represented by asphaltene–resin complexes consisting of asphaltenes, as well as solid and high-melting resins, which are distributed in a dispersive medium of maltenes, composed of oils and low-melting resins [8]. According to this theory, the structure and properties of bitumen are determined not only by the chemical compositions of the components but also by the size of the associates, which influence the molecular, supramolecular, inductive (topological), and colloidal-dispersion levels—highlighting the importance of the entire complex of structural formation phenomena.

However, it is important to note that when adding additional components to bitumen, in this case, crumb rubber (CR) and plasticizers form more complicated structures of organized polydispersive materials. These represent a complex statistical ensemble of macro- and microsized components with varying physical and mechanical properties, granulometric composition, and geometric shapes, distributed within a multiphase medium and interacting with each other [9].

The mechanism of interaction between the particles of the crumb rubber and the bitumen binder plays a major role in determining the quality and performance properties of the crumb-rubber-modified binder. To establish the influence of crumb rubber on the structures of complex systems, modern scientific and technical sources describing the interaction mechanism of crumb-rubber-modified binder components were studied and analyzed [10,11,12,13,14,15,16,17,18,19,20,21,22,23,24,25,26,27,28,29,30,31,32,33,34,35,36,37,38,39,40].

It was found that in the process of interaction between CR and bitumen, the following two main mechanisms occur: swelling of crumb rubber particles [10] and their physical or chemical destruction (devulcanization and depolymerization) [11,12]. Rubber swelling is a physical process of aromatic substance adsorption and partial diffusion. Typically, the swelling process is followed by destruction. The chemical destruction of rubber causes the cross-linking to break down. Most often this involves sulfur, less often resin or peroxide [13,14]. Reorientation of the molecules occurs forming new substances. In the literature, researchers use the term “rubber dissolution” to describe the state of rubber particles in bitumen during their interaction [15,16]. Rubber dissolution is measured by extracting rubber particles from the binder matrix; the portion of rubber particles that passes through a fine sieve (usually 75 µm) is considered the dissolved part [11,17]. This definition of the term “dissolution” is inaccurate and does not determine whether the chemical destruction of rubber has occurred. It should be noted that swelling and complete dissolution of rubber are at two opposite ends of the spectrum of interactions between bitumen and rubber, depending on the interaction conditions.

Uncrosslinked polymers easily swell because of the action of compatible solvents and then undergo partial dissolution [18,19]. The dissolution of an uncrosslinked polymer in a solvent involves the following two phenomena: swelling caused by solvent diffusion and chain disentanglement [11,20]. Polymers with a crosslinked structure, where bonds exist among chains or segments, can swell by absorbing solvents, but the dissolution process is minimal. For polymers with a crosslinked network structure, such as crumb rubber derived from ground used tires, a characteristic property is limited swelling (or partial dissolution) [11,16]. When rubber is mixed with bitumen at high temperatures, light fractions of the bitumen diffuse into the crumb rubber, causing it to expand in volume, which can be described as swelling [20]. The change in the volume of the rubber particles and the formation of a gel layer adjacent to the rubber–bitumen interface reduces the distance between the RC particles and alters the ratio of components in the remaining bitumen, leading to increased stiffness of the composite material [11,21].

Polymer destruction is the process of breaking down the rubber network under harsh interaction conditions, such as excessively high mixing temperatures, high shear forces, and prolonged mixing times. Under these conditions the sewn polymer mesh of the crumb rubber is destroyed by high thermal energy and shear energy [22]. The first process of devulcanization involves the breaking of disulfide bonds (SS) and carbon–sulfur bonds (C–S); that is, the cross-links breaks. This is why a sulfoxide odor can be detected during the preparation of crumb-rubber-modified binder at high temperatures. Next, depolymerization occurs, breaking the carbon–carbon (C–C) bonds in the main chain, thereby reducing the average molecular weight of the rubber [11]. Chemical destruction of the polymer network in crumb rubber negatively affects the mechanical properties of bitumen binders [23,24] but positively influences improvements in crumb-rubber-modified binders’ storage stability [25].

It is also important to note that crumb rubber derived from ground used tires contains a mixture of various components—plasticizers, carbon black, and inorganic fillers—which are released into the bitumen matrix during interactions under high temperatures and mixing [26]. Their influence on the properties of the bitumen binder cannot be ignored—it has been noted that these components significantly affect the aging and rheological properties of rubber–bitumen binders [27,28].

Thus, according to modern technology, the mechanism of interaction between crumb rubber and bitumen can be described in the following three main stages [12,29]:

(1) The first phase: swelling, whereby RC particles begin to increase in volume, absorbing light fractions of the bitumen, and a gel-like layer forms adjacent to the bitumen oil fractions–RC interface;
(2) The second phase: The subsequent swelling and beginning of destruction occur, when the swelling of the crumb rubber particles continues. Chemical destruction of crumb rubber occurs due the breakdown of the vulcanized polymer mesh. As a result, the swollen crumb rubber particles break down into smaller pieces;
(3) The third phase: destruction and complete dissolution, whereby the already devulcanized RC particles undergo further polymer network breakdown until the RC particles are completely dissolved in the bitumen matrix, resulting in a homogeneous modified bitumen binder.

The degree of swelling and destruction of crumb rubber in bitumen plays a crucial role in the formation of the performance properties of crumb-rubber-modified binder [30]. Therefore, controlling the degree of RC swelling and destruction by adjusting the conditions for producing crumb-rubber-modified binder has attracted the attention of many researchers seeking to obtain crumb-rubber-modified binder with improved performance properties.

To study the mechanism of RC particle swelling and devulcanization in bitumen binder, a method involving the stepwise extraction of swollen rubber was used [31], allowing the CR–bitumen interaction zone to be divided into four layers for sequential investigation. Gel permeation chromatography results reveal that bitumen fractions with lower molecular weights were absorbed into the deeper layers of the swelling rubber. It was also noted that there was minimal absorption of bitumen fractions containing carbon–oxygen (C-O) bonds. In a modeling study [21], dibutyl phthalate was selected to simulate the light components in bitumen, and a soaking test was conducted with rubber. A use test was conducted for the impregnation of rubber. The gas chromatography-mass spectrometry results led to the conclusion that chemical reactions occur in the “rubber–dibutyl phthalate” system, including light component absorption, rubber decomposition with chain breakage, and the formation of new compounds. The study [21] also confirmed that changes in viscosity can reflect the physical–chemical properties of RC particles in bitumen. In particular, the viscosity first increased and then decreased after reaching a peak value when the crumb rubber content was more than 20 wt.% and the mixing time was more than 90 min. It was shown that both high temperatures and prolonged times are causes of CR polymer chain destruction, leading to a decrease in viscosity. In conclusion, it can be noted that the viscosity of crumb-rubber-modified binder gradually increases, reaching an equilibrium as the CR swells, and then decreases with an increase in the degree of degradation and the amount of degraded CR.

The studies and methods considered have both obvious advantages [3,32] and disadvantages for binders modified with crumb rubber [33,34]. To eliminate these disadvantages, several promising methods have been developed, generally involving CR activation, which consists in the surface destruction of crumb rubber due to shear effects [10,35,36], exposure to ionizing radiation [37], or treatment with a devulcanizer [12,38,39,40].

However, these methods still face the problem of dispersing the crumb rubber in the bitumen. Because the melting process results in significant increases in particle surfaces and/or changes in the surface wettability of the crumb rubber particles, this subsequently leads to the formation of aggregates composed of the unwetted particles, which has an intense elastic aftereffect, leading to cracking, especially during low-temperature operation, and, therefore, premature destruction of road surfaces [41,42]. It should also be noted that the lack of knowledge about crumb rubber compositions and their stability and compatibility with plasticizers, as well as the influence of various factors on the efficiency of the rubber devulcanization process and its subsequent connection with bitumen, leads to variability in research results and ambiguity in the recommended technological modes. Current problems include a lack of studies aimed at establishing a mechanism for the interaction between plasticizers and crumb rubber (macromolecular or supramolecular). Moreover, a lack of understanding of the interaction process between hydrocarbon plasticizers and devulcanized crumb rubber prevents the effective control of its dissolution process to obtain dispersed systems with specified property parameters and stability.

According to regulatory documents enforced in the Russian Federation, one of the most important indicators for crumb-rubber-modified binder is crumb rubber’s solubility in a bitumen volume of at least 99%; otherwise, such a modified binder cannot be used for road surface construction. Currently, there are no crumb-rubber-modified binders in our country that meet these requirements. Thus, this work is devoted to the study of crumb rubber compositions and their stability, as well as the determination of the compatibility between the crumb rubber and plasticizers and investigation of the mechanism of interaction between them (macromolecular or supramolecular). New data will allow for the formulation of a hypothesis for the formation of a sustainable and thermodynamically stable structure of crumb-rubber-modified binder. This will also contribute to the increased stability and durability of asphalt concrete while simultaneously reducing construction costs and improving the environmental aspect of used tire disposal.

## 2. Materials and Methods

### 2.1. Raw Materials

The following materials derived from waste rubber products were considered:

(1) Activated crumb rubber from large-sized tires (NCR LSTs) with a fraction size of 0.3–0.6 mm produced by “NIINTC DorNau” LLC, Nizhny Novgorod, Russia. Obtained by mechanically grinding chips from large-sized tires (tires with a rim diameter of 50 cm or more, intended for special-purpose heavy machinery operating under harsh conditions) through tearing and abrasive impact at ambient temperature on an Amandus Kahl equipment production line.
(2) Activated crumb rubber from large-sized tires (ACR LSTs) with a fraction size of 0.3–0.6 mm produced by “NIINTC DorNau” LLC, Nizhny Novgorod, Russia, obtained by activating NCR LSTs using a “Leistritz” industrial extruder (Leistritz extrusion technology, Nuremberg, Germany) through high-temperature shear impact at 140 °C.
(3) Crumb rubber (CR CRP 0.5)* produced by “Chekhov Regenerate Plant” LLC, Moscow, Russia, obtained by crushing and grinding pneumatic tires from passenger vehicles with step-by-step removal of textile, synthetic, and metal cords on an “Eldan” equipment production line.

**Figure 2:**
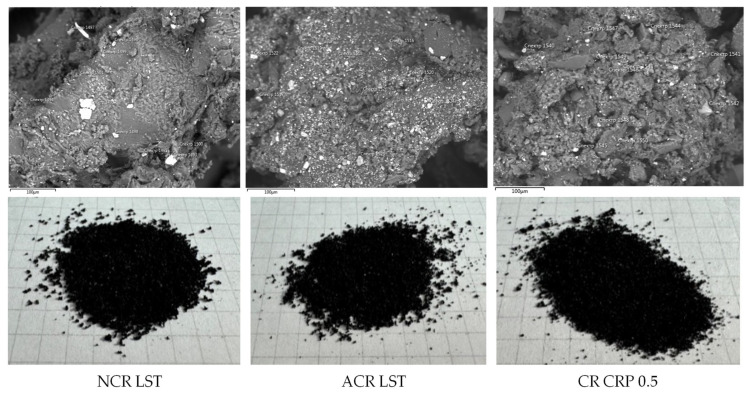
Investigated samples of crumb rubber.

The appearance (1 cell = 0.5 × 0.5 cm) and morphostructural features of the studied crumb rubber samples are shown in Figure 2. The microstructural photographs were obtained using a JEOL JSM-IT300LV scanning electron microscope (JEOL, Tokyo, Japan).

The following hydrocarbon plasticizers were considered:

(1) Residual oil extract (ROE), produced by “LUKOIL-Volgogradneftepererabotka” LLC, Volgograd, Russia. The technological process used to produce the residual extract involves the following: residual oil from the vacuum section of the atmospheric-vacuum column undergoes propane de-asphalting, where it is separated into asphalt and de-asphalted oils. The de-asphalted oil is then subjected to selective purification, resulting in a raffinate, which is used to produce diesel fuel, and a residual selective purification extract.
(2) Waste industrial oil (WIO), obtained after use in an ammonia production unit. The waste industrial oil was provided by the “Azot” branch of “UralChem” JSC in Berezniki, Perm Krai, Russia.
(3) Purified waste frying oil (PWFO) produced by “Promekoglobal” LLC, Moscow, Russia. The production process involves filtering waste frying oil using cyclones to remove mechanical impurities and water. The purified waste frying oil consists of organic non-lauric mono-, di-, and triglycerides and distilled free fatty acids.
(4) Plasticizer for Polymer Modified Binders “RN-21” produced by “TK RIMINVEST” LLC, Nizhny Novgorod, Russia. The production process involves compounding various petroleum feedstocks (petroleum oils) and residues from petroleum refining processes.

The physical properties of the studied plasticizers are presented in Table 1.

To prepare the crumb-rubber-modified binder, road bitumen of grade BND 70/100, produced by “LUKOIL-Nizhegorodnefteorgsintez” LLC, Nizhny Novgorod, Russia, was used. The bitumen was tested for compliance with the requirements of the Russian State Standard GOST 33133-14 [43]. The results of laboratory tests on the physical and mechanical properties of the bitumen are presented in Table 2.

### 2.2. Methods

#### 2.2.1. Object under Study: Crumb Rubber

##### Determination of the Mass Ratio of the Polymer Component in the Crumb Rubber

The analyzed samples of crumb rubbers were obtained by grinding vehicle tires, which are known to include various fillers and plasticizers. Therefore, in the first stage of the research, the mass ratio of the polymer component to other rubber components was studied using the acetone extraction method, as outlined in Russian State Standard GOST 545500-2011 [44]. The method is used to determine the total mass proportion of substances extracted from rubber. The method allows for obtaining the total numbers of tar and fatty acids, soap, oils, resins, antioxidants, and other non-polymer chain-related substances. The mass fraction of rubber hydrocarbons can be determined by subtracting from a hundred the total number of substances extracted.

##### Determination of the Stability of the Composition of the Crumb Rubbers

**Figure 3:**
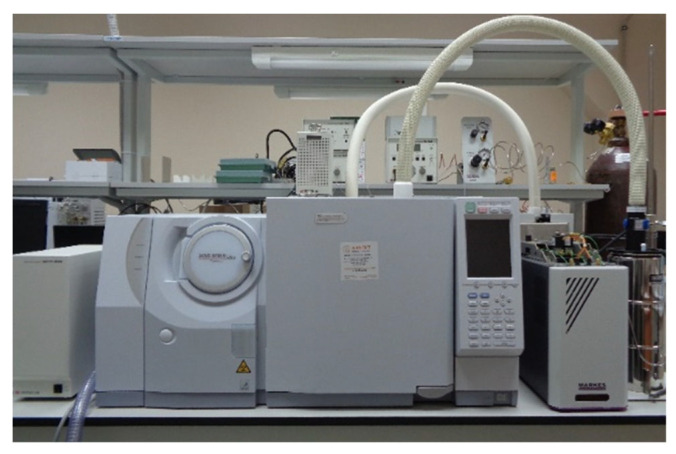
GCMS-QP2010 gas chromatography-mass spectrometer with a pyrolyzer and thermodesorber.

There are concerns about the stability of secondary resources, as it is generally believed that the stability of production waste cannot be controlled. To investigate this issue, the types of polymers in the analyzed crumb rubber samples were identified and compared with samples from other batches and different fractions after extracting soluble components using pyrolytic gas chromatography-mass spectrometry with a GCMS-QP2010 gas chromatography-mass spectrometer equipped with a pyrolyzer and thermodesorber (manufactured by Shimadzu USA Manufacturing, Inc., Canby, OR, USA) (Figure 3).

This method allows for qualitative analysis of organic nonvolatile components using pyrolytic gas chromatography-mass spectrometry by analyzing the thermal decomposition products at 600 °C.

##### Study of the Surface Characteristics and Chemical Composition of the Crumb Rubbers

**Figure 4:**
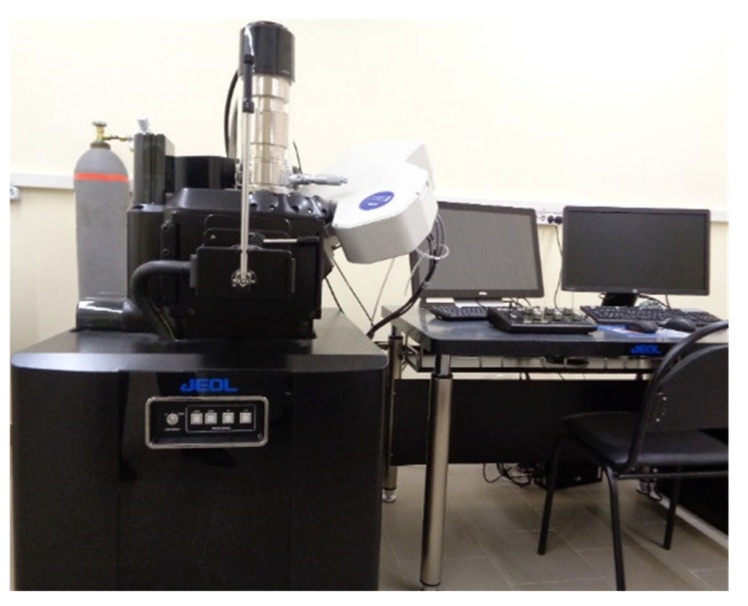
JEOL JSM-IT300LV scanning electron microscope with energy- and wave-dispersion attachments.

It is believed that the interaction intensity of crumb rubber with the bitumen base fractions depends on the surface characteristics of the crumb rubber. To determine these surface characteristics, the microstructure was examined using scanning electron microscopy combined with semi-quantitative elemental analysis. Images with nanometer resolutions were obtained and surface elemental analysis of the materials was conducted using a JEOL JSM-IT300LV scanning electron microscope with energy- and wavelength-dispersive attachments (JEOL Ltd., Tokyo, Japan) (Figure 4). The photographs were taken after examining no fewer than 100 areas of a sample. The location for each image was chosen to provide the clearest and most accessible information.

##### Determination of the Thermodynamic Properties of the Crumb Rubbers

**Figure 5:**
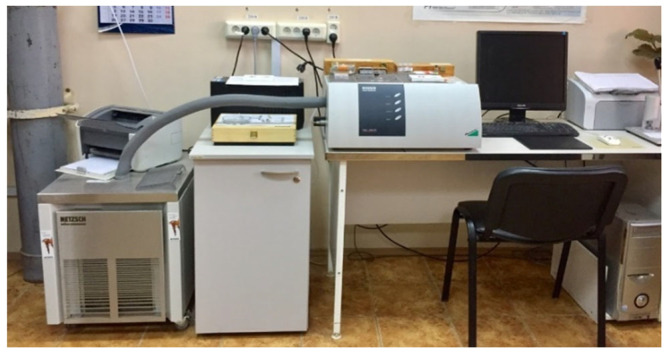
Differential scanning calorimeter DSC 204 F1 Phoenix.

When developing a thermodynamically stable structure of crumb-rubber-modified binder, it is important to determine the thermodynamic properties of the initial crumb rubbers, such as the decomposition temperature. The testing was conducted according to Russian State Standard GOST R 55134-2012 (ISO 11357-1:2009) [45] using the differential scanning calorimetry (DSC) method, with the application of negative temperatures on the DSC 204 F1 Phoenix differential scanning calorimeter (Netzsch, Hanau, Germany) (Figure 5). This method is designed to detect the presence of thermal effects in the tested materials by measuring the temperature dependence of the difference between the heat flows of the sample and the reference.

##### Determination of the Nature of the Polymer Component in the Crumb Rubbers

By applying pyrolytic gas chromatography-mass spectrometry with a GCMS-QP2010 gas chromatography-mass spectrometer equipped with a pyrolyzer and thermodesorber (manufactured by SHIMADZU USA MANUFACTURING, INC, USA) (Figure 3), the nature of the polymer component in the studied crumb rubber samples was determined.

#### 2.2.2. Object under Study: Crumb Rubbers and Hydrocarbon Plasticizers

##### Determination of the Compatibility of the Hydrocarbon Plasticizers with the Studied Crumb Rubbers

The compatibility of the hydrocarbon plasticizers with the studied crumb rubber samples was assessed using thermodynamic methods and techniques developed in this field because of the complexity and diversity of the phase compositions of the crumb rubbers and hydrocarbon plasticizers when predicting the compatibility of hydrocarbon plasticizers and the possibility of optimizing the experiment. The authors of this study previously published an article that reviews some of the parameters related to the compatibility of “hydrocarbon plasticizer–crumb rubber” in dispersed systems [2]. However, it is worth noting that they were considered with some assumptions, which this study aims to eliminate. Two chemical compatibility parameters were studied (Traxler’s dispersibility coefficient and the mass ratio of paraffin-naphthenic compounds to asphaltenes), which consider only the characteristics of the hydrocarbon medium. Additionally, the Hildebrand solubility parameter was calculated based on the evaporation energy criterion, which considers the parameters of the entire dispersed system and characterizes the intensity of intermolecular interactions in the substance. This parameter is numerically equal to the energy required to separate molecules to the distance at which the interaction forces can be neglected. However, it should be noted that in the previous calculation, an assumption was made: that the absolute volume of the substance was used instead of the molar volume.

Therefore, in this study, corrections were made in the calculation of the Hildebrand compatibility parameter based on the evaporation energy criterion [2]. At the same time, the condition corresponding to dissolution—the minimum Gibbs free energy—remains valid. This condition can be achieved when the solubility parameter of the plasticizer, *δP*, and the modifier, *δM*, are equal.

(1) $$\delta P=\sqrt{\frac{E}{V_{m}}}=\sqrt{\frac{kT_{FP}}{V_{m}}};\;\delta M=\sqrt{\frac{E}{V_{m}}}=\sqrt{\frac{kT_{B}}{V_{m}}},$$

where *δP* and *δM*—solubility parameters of the plasticizer and modifier, respectively, (J/cm^3^)^0.5^; *E*—evaporation energy, J/mole; *V_m_*—molar volume of the substance, cm^3^/mole; *k*—a constant equal to 89.2 J/(mole × K); *T_FP_*—flash point of the plasticizer, K; and *T_B_*—modifier burnout temperature, K.

The molar volumes of the hydrocarbon media were calculated using the following formula:

(2) $$V_{m}=M/\rho ,$$

where *M*—molar mass, g/mole, and ρ—density, g/cm^3^.

Various petroleum fractions were considered for the studied hydrocarbon media. The relationship between the molecular weight (numerically equal to the molar mass) and the relative density of the petroleum fractions can be determined using Craig’s formula [46], as follows:

(3) $$M=\frac{44.29\rho_{15}^{15}}{1.03-\rho_{15}^{15}},$$

where $\rho_{15}^{15}$—relative density and dimensionless value (where 15—the standard temperature of the petroleum products and water, degrees Celsius, °C), which can be found using the following formula:

(4) $$\rho_{15}^{15}=\rho_{4}^{20}+\frac{0.0035}{\rho_{4}^{20}},$$

where $\rho_{4}^{20}$—relative density equal to the ratio of the density of the petroleum products at 20 °C to the density of water at 4 °C.

Since the density of water at this temperature is 1 g/cm^3^, the numerical values of the relative and absolute densities coincide in this case. Therefore, the formula for calculating the molar volume takes the following form:

(5) $$V_{m}=\frac{44.29\rho_{15}^{15}}{(1.03-\rho_{15}^{15}){\times \rho }_{4}^{20}},$$

We also compared the values obtained for the studied “hydrocarbon plasticizer–crumb rubber” dispersed system using Hildebrand compatibility parameters based on the evaporation energy criterion and the Hildebrand parameters based on the thermodynamic criterion for surface tension. According to Stefan’s rule, a relationship is established between the enthalpy of evaporation, molar volume (or molar mass and density), cohesion energy density, and surface tension. Based on Stefan’s rule, Hildebrand and Scott [47] expressed the dependence of the surface tension of liquids on the enthalpy of evaporation as follows:

(6) $$\sigma_{L}=0.13\;∆H_{vap}{V_{m}}^{\frac{2}{3}}$$

where $\sigma_{L}$—surface tension of the liquid, J/m^2^; $∆H_{vap}$—enthalpy of evaporation, kJ/mole; and $V_{m}$—molar volume, cm^3^/mole.

From this, the enthalpy of evaporation, characterized as the amount of heat required to convert a substance from a liquid state to a gaseous state at a constant pressure and temperature, can be expressed as follows:

(7) $$∆H_{vap}=\frac{\sigma_{L}}{0.13\;{V_{m}}^{\frac{2}{3}}}$$

By substituting this equation into Formula (1), we obtain the following formula for determining the Hildebrand solubility parameter based on the thermodynamic criterion:

(8) $$\delta =\sqrt{\frac{\sigma_{L}\;}{0.13{V_{m}}^{\frac{5}{3}}}}$$

Similar formulas are also known. For example, Hildebrand and Scott [48] proposed the following empirical relationship for determining compatibility:

(9) $$\delta_{T}={4.1\left(\frac{\sigma_{L}}{{V_{m}}^{\frac{1}{3}}}\right)}^{0.43}$$

However, it should be noted that this equation is quite unsatisfactory, because it is dimensionally inconsistent in the exponent, although it often provides satisfactory results. Later, Birnbaum [49] refined the relationship (Formula (9)) based on an extensive database, as follows:

(10) $$\delta_{T}={3.741\left(\frac{\sigma_{L}}{{V_{m}}^{\frac{1}{3}}}\right)}^{\frac{1}{2}}$$

Subsequently, Formula (10) was used to calculate the solubility parameters of the plasticizer and modifier.

In [50,51], Patterson presents results comparing predictions from a theory based on the principle of corresponding states and a theory using solubility parameters to estimate solubility. Based on this comparison, a value *X*_1_ was proposed, equal to the following:

(11) $$X_{1}=\beta +\frac{V_{1}}{RT}{\left(\delta_{1}-\delta_{2}\right)}^{2}$$

where *V_m_*—molar volume of the component, cm^3^/mole; *T*—interaction temperature, *K*; *R*—universal gas constant, equal to 8.314 J/(mole × K); *δ*_1_ and *δ*_2_—solubility parameters of components 1 and 2; and β—correction factor equal to 0.34 and characterizing the differences in the structure of polymers in solution from quasi-spherical molecules.

Formula (11) was used to assess the solubility of crumb rubber in the studied plasticizers.

Additional factors determining the compatibility of the plasticizer and modifier include the following:

- Burshtein’s interaction parameter (|X|), which is determined by the following formula [52]:
  (12) $$|X|=\frac{0.002226V_{m}+0.1351-\frac{V_{m}}{T_{m}}}{0.1351}$$
  where |X|—plasticizer and modifier compatibility parameter; $T_{m}$—temperature of the interaction of the crumb rubber and the plasticizer, *K*; and $V_{m}$—molar volume of the plasticizer, cm^3^/mole. According to theoretical and experimental data [52], it has been established that depending on the value of parameter |X|, plasticizers are divided into the following two groups: compatible—when |X| < 0.55; partially or incompatible—when |X| > 0.55. Therefore, for an optimal plasticizer, it is necessary to aim for the Burshtein’s interaction parameter to be <0.55 → min.
- Traxler’s dispersibility coefficient: The higher the coefficient, the more aromatic fractions are present in the plasticizer, which have a high affinity for crumb rubber and are able to interact with the rubber polymer molecules. Polymer molecules from crumb rubber swell. Resin helps to stabilize the swelling process. Resin forms a solvate shell around the swelling polymer particles. The Traxler’s dispersibility coefficient is determined by the following formula:
  (13) $$TDC=\frac{Ar+R}{PN+As}$$
  where *Ar*—aromatics (including light aromatics, medium aromatics, and heavy aromatics), %; *R*—resins, %; *PN*—paraffin naphthenic, %; and As—asphaltenes, %.
- The mass ratio of paraffin-naphthenic compounds to asphaltenes: the higher the PN:As ratio, the better the dissolving ability, the lower the viscosity of the plasticizer, and presumably, the faster the processes of rubber swelling and dispersion.

**Figure 6:**
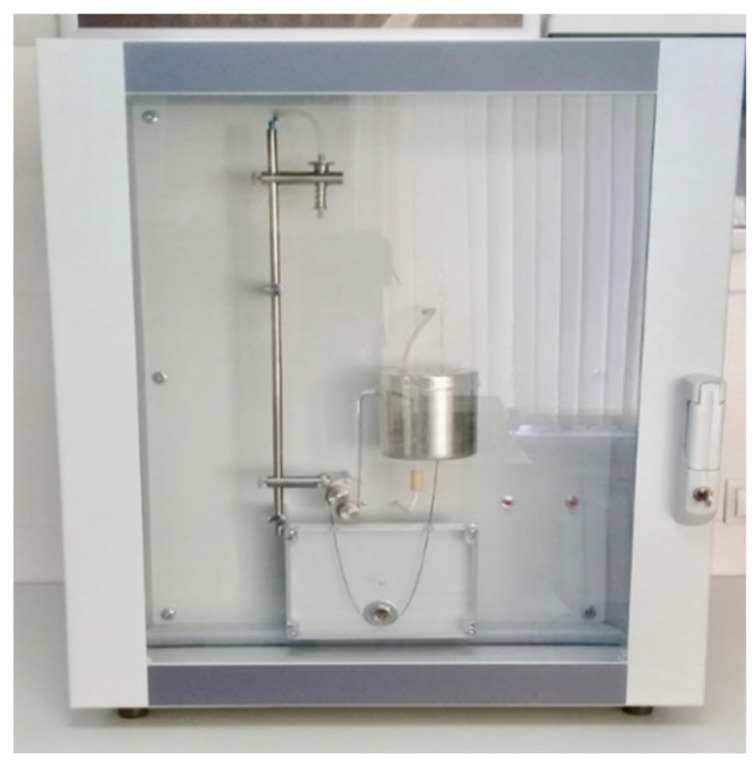
Gradient M unit, designed by GUP INHP RB.

To calculate the semi-empirical parameters, the group hydrocarbon composition and the surface (interfacial) tension of the plasticizers were determined. The group hydrocarbon composition was determined using liquid-adsorption chromatography with gradient displacement on the “Gradient-M” laboratory setup according to the method of GUP INHP RB (Figure 6). The essence of the method lies in the stepwise gradient displacement separation of high-boiling heavy petroleum products into 7 groups, followed by their registration with a thermal conductivity detector.

**Figure 7:**
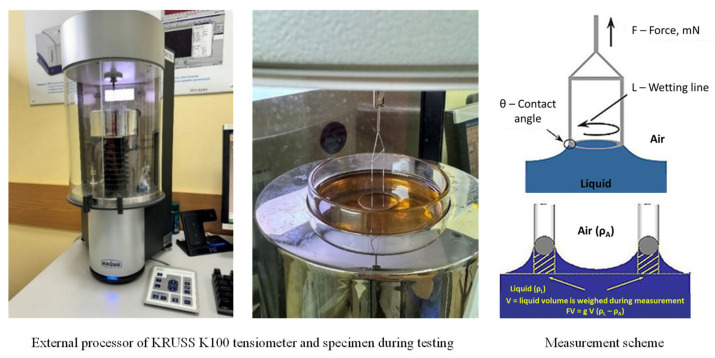
Measurement of surface tension using the ring break-off method.

The surface (interfacial) tension of the hydrocarbon plasticizers was determined using a processor tensiometer from KRUSS, employing the ring detachment method (Figure 7). This method is based on measuring the maximum force (F) required to detach a ring with a known geometry (wetting length, L), made from a well-wettable material (wetting angle θ = 0°). As the ring is lifted, the liquid tends to flow off it, leading to a gradual thinning of the liquid film and the detachment of the ring.

#### 2.2.3. Object under Study: Crumb-Rubber-Modified Binder

##### Mathematical Experiment Design

To obtain experimental–statistical models of the influence of the main formulation and/or technological factors on the properties of the research object and to make them suitable for subsequent multicriteria optimization of the crumb-rubber-modified binder production process, a mathematical experiment design method—a composite two-factor plan—was used. In the development of the crumb-rubber-modified binder, the bitumen content was constant and set at 100%. The factors chosen were the concentration of crumb rubber (X1) and the concentration of the hydrocarbon plasticizer (X2). The selected planning parameters are shown in Table 3.

The planning matrix is presented in Table 4.

##### Technology Used in the Preparation of the Crumb-Rubber-Modified Binder

The process of preparing crumb-rubber-modified binder (CRMB) was as follows. In the first stage, the required amounts of raw materials (crumb rubber, hydrocarbon plasticizer, and bitumen binder) were weighed. The bitumen binder was preheated to 160 °C in a drying oven. Next, a bitumen base for modification was prepared by mixing the heated bitumen binder with the plasticizer, using a high-shear mixer Silverson L5M-A with a heat control sensor immersed in the container, and the system was heated to 175 °C and had a stirring speed of 2000 rpm. Once the temperature reached 175 °C, the stirring speed was increased to 5000 rpm, the crumb rubber was added to the bitumen base, and the components of the system were mixed for 40 min. In the next stage, the prepared CRMB underwent a maturation process by mixing it with an IKA EUROSTAR 100 digital stirrer (IKA Werke, Staufen, Germany) at a speed of 300 rpm at 175 °C for 3 h. To complete the rubber network formation process, the maturation was continued in a drying oven at 175 °C for 18 h, which can also be considered a simulation of the actual production process. Laboratory processing of the modified binder in the oven modeled the real transportation of the binder to the end consumer.

##### Determination of the Homogeneity, Solubility, and Delamination of the Obtained Crumb-Rubber-Modified Binder

The homogeneity of the obtained crumb-rubber-modified binder was determined according to Russian State Standard GOST R 52056-2003 [53]. The essence of this method lies in visually determining the homogeneity of the modified binder using a glass rod. Before testing, the modified binder sample should be heated to a temperature 10 °C higher than the preparation temperature of the modified binder and stirred for 5–6 min. The glass rod was immersed in the prepared sample for 3–4 s, then removed, and the flow of the modified binder off the rod and the state of the binder film on its surface were visually evaluated. The modified binder should flow off the rod evenly, and there should be no clumps, lumps, or granules on its surface. The homogeneity of the crumb-rubber-modified binder was determined by comparing the results of three tests. If two out of three tests had positive results, the modified binder was considered to have passed the homogeneity test.

**Figure 8:**
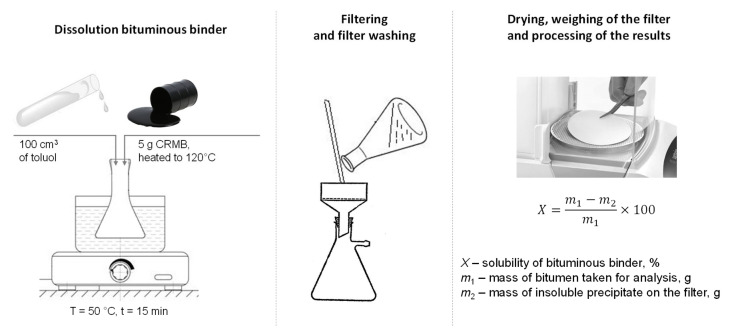
Determination of the solubility of the CRMB according to Russian State Standard GOST 33135-2014.

The solubility of the obtained crumb-rubber-modified binder was determined according to Russian State Standard GOST 33135-2014 [54]. This method involves determining the solubility degree of the crumb-rubber-modified binder in an organic solvent, specifically toluene. Five crumb-rubber-modified binder samples were selected and heated in the drying oven at a temperature of 120 °C. Further, the heated CRMB was dissolved in 100 cm^3^ toulene in a heating flask in a water bath (50 °C) while mixing. The flask was then covered and left for 15 min. After, the modified binder solution was filtered through a dried, ash-free double filter placed in a funnel. The filter pore size was 2 microns. The residue in the flask was washed onto the filter with clean solvent preheated to 50 °C. Upon completion of the filtration, the filter with the sediment was washed with the preheated solvent. After washing, the filter with the sediment was transferred to a weighing cup and dried with the lid open for at least 20 min in a drying oven at 20 °C higher than the solvent boiling temperature. The weighing cup was then covered with a lid, cooled in a desiccator for 30 min, and weighed. A schematic of the testing method is shown in Figure 8.

The solubility of the CRMB was calculated by the following formula:

(14) $$X=\frac{m_{1}-m_{2}}{m_{1}}\times 100,$$

where *X*—solubility of the modified binder, %; *m*_1_—mass of the modified binder taken for analysis, g; and *m*_2_—mass of the insoluble residue on the filter, g.

**Figure 9:**
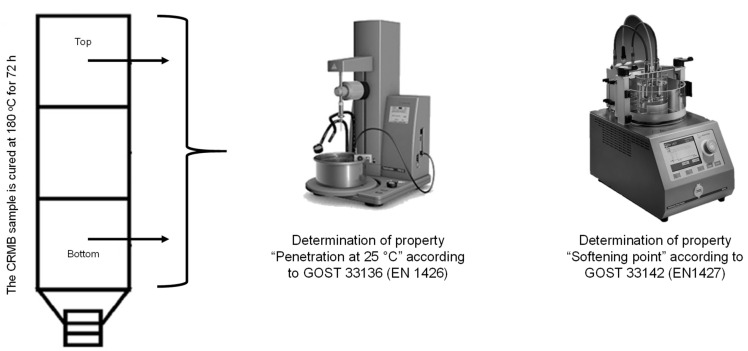
Determination of the delamination of the CRMB according to Russian State Standard GOST EN 13399-2013.

The delamination of the obtained crumb-rubber-modified binder during storage was determined according to Russian State Standard GOST EN 13399-2013 [55]. The essence of this method is that a homogeneous sample of the modified binder was kept in a tube (a vertical container with a height of 100–120 mm) for three days at a temperature of 180 °C. After cooling, the sample was cut into three equal parts, and the properties of the top and bottom parts were evaluated. In the Russian Federation, the properties “Penetration depth of a needle at 25 °C”, according to Russian State Standard GOST 33136 (EN 1426) [56], and “Softening point by the ring-and-ball method”, according to Russian State Standard GOST 33142 (EN 1427) [57], were determined for the top and bottom parts, and the difference in the values between the top and bottom parts was calculated. The crumb-rubber-modified binder was considered stable if the difference in the “Softening point by the ring-and-ball method” was no more than 3 °C and for the “Penetration depth of a needle at 25 °C” at no more than 20, 0.1 mm. A schematic of the testing method is shown in Figure 9.

## 3. Results and Discussion

### 3.1. Object under Study: Crumb Rubber

#### 3.1.1. Determination the Mass Ratio of the Polymer Component in the Crumb Rubber

**Figure 10:**
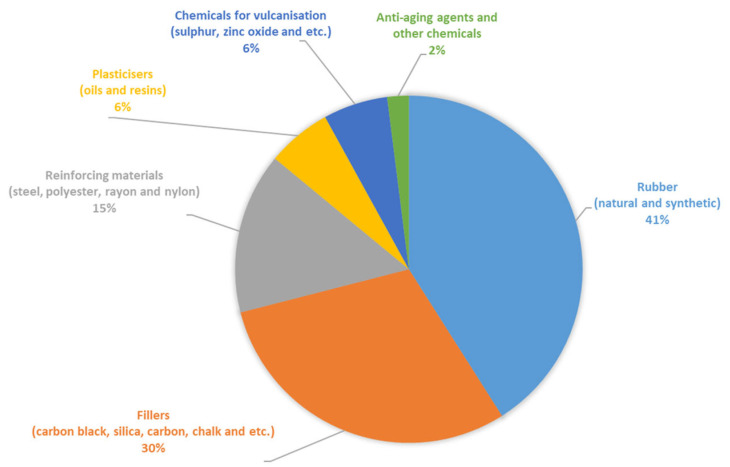
Approximate component composition of the tires.

The chemical composition of the materials used from rubber waste products—crumb rubbers from worn tires—included a wide range of polymers and other components. The formulation of the rubber mixtures used in tire manufacturing is a commercial secret of the manufacturers and can only be provided in approximate ranges. Figure 10 schematically shows the approximate component composition of tires based on the analysis of publicly available information sources.

During the production of crumb rubber, the waste tires were cleaned of reinforcing materials and fillers (e.g., textile and metal cords). However, the exact percentages of the components in the crumb rubbers were not determined during their production. Therefore, determining the composition of the crumb rubbers used in the production of the crumb-rubber-modified binder involved the quality control of materials into the production system. To achieve this, the mass ratio of the polymer component to other rubber components was studied and determined using the acetone extraction method, with the results presented in Table 5.

Based on the research results (Table 5), it was established that the average mass and range of variation fraction of the rubber hydrocarbons in the studied samples was 93.3 ± 1.8%. Such a high content of rubber hydrocarbons allows for the exclusion of other chemical substances present in the studied samples of crumb rubbers in the initial stage of the research.

#### 3.1.2. Determination of the Stability of the Composition of the Crumb Rubbers

**Figure 11:**
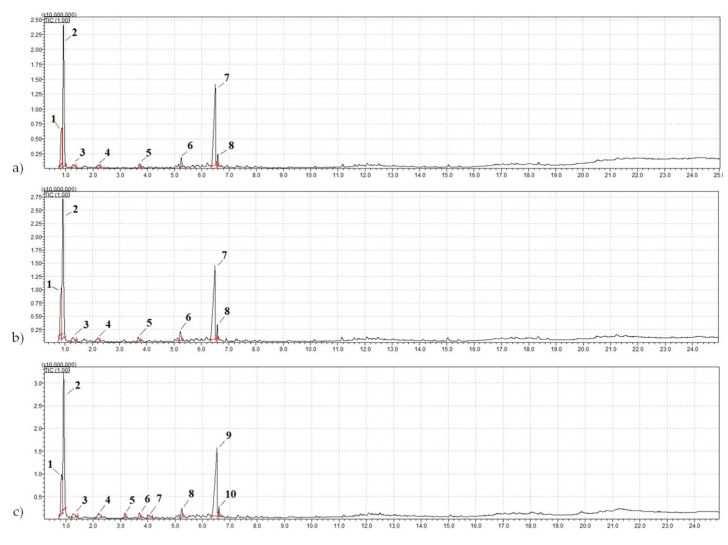
Mass chromatogram of the pyrolysis-GCMS products of the samples: (**a**) NCR LST; (**b**) ACR LST; (**c**) CR CRP 0.5.

Secondary resources often raise questions regarding the stability of the raw materials, particularly in terms of the range of their characteristic variations. To investigate this issue, pyrolytic gas chromatography-mass spectrometry (pyrolysis—GCMS) was conducted. Additionally, to establish the stability of the components in the crumb rubbers, samples from other fractions and batches were collected. The mass chromatograms of the pyrolysis-GCMS products of the crumb rubber samples are shown in Figure 11. The results of the qualitative analysis of the organic nonvolatile components using pyrolytic gas chromatography-mass spectrometry based on the products of their thermal decomposition (600 °C) are presented in Table 6.

Based on the research results (Table 6), it was established that the samples of crumb rubber (CR) of the same type, but taken from different batches and various fractions, contained identical polymers, indicating the stability of the crumb rubber composition.

#### 3.1.3. Study of the Surface Characteristics and Chemical Composition of the Crumb Rubbers

**Figure 12:**
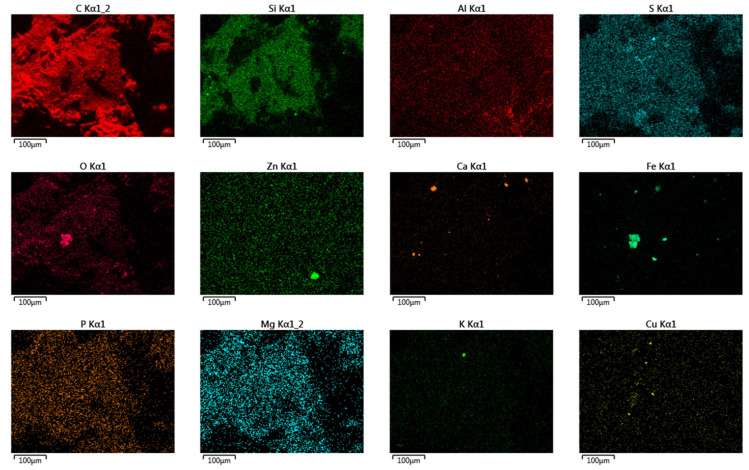
Distribution of individual elements on the surface of crumb rubbers (NCR LST used for example).

The results of the crumb rubbers’ analysis in their original form using electron microscopy combined with semi-quantitative elemental analysis (using a JEOL JSM-IT300LV scanning electron microscope with energy- and wavelength-dispersive elemental analyzers) are shown in Figure 12. A summary of the elements and their contents on the surface of the crumb rubber samples is presented in Table 7.

As a result of the surface analysis of the provided samples using scanning electron microscopy in combination with semi-quantitative elemental analysis, the following elements were detected on the surfaces of all samples, in addition to carbon: oxygen, aluminum, silicon, sulfur, calcium, iron, and zinc. The sample ACR LIST contained ferrum, cuprum, and potassium, all CR samples contained magnesium, and the CR CRP 0.5 sample contained potassium and chlorine.

#### 3.1.4. Determination of the Thermodynamic Properties of the Crumb Rubbers

Using differential scanning calorimetry, the thermodynamic properties of the initial CR samples, such as degradation destruction temperatures were determined and are presented in Table 8.

The glass transition temperature indicates the elasticity and flexibility of the polymer products at a given temperature. In other words, the lower this temperature, the wider the temperature range in which the CR samples maintain their operational characteristics. As shown in Table 8, the sample ACR LST had the lowest glass transition temperature at 9.59 °C, while for the nonactivated samples NCR LST and CR CRP 0.5, the temperatures were higher by 34% and 100%, respectively. The decomposition (complete degradation) temperatures for all of the studied crumb rubber samples were within a range of 300 to 310 °C. Thus, the ACR LST sample is the most attractive in terms of temperature range for maintaining its properties.

#### 3.1.5. Determination of the Polymer Nature in Crumb Rubbers

By conducting pyrolysis-gas chromatography-mass spectrometry on their thermal decay products, the natures of the polymer components in the crumb rubbers were established. The natures of the polymer components in the crumb rubbers (Table 9) were determined from the data obtained (Table 6).

It was found that both the nonactivated and activated crumb rubbers from large-sized tires were based on butadiene rubber, while the CR CRP 0.5 was based on butadiene–styrene rubber. These rubbers belong to elastomers, and the properties are listed in Table 10.

It is known that butadiene–styrene rubber has increased viscosity and rigidity, requiring additional effort and energy for its grinding and homogenization within the system. Butadiene rubber, on the other hand, exhibits high elasticity over a wide temperature range. Additionally, according to the literature [35,36], activated crumb rubber interacts better with the components of crumb-rubber-modified binder. Therefore, considering the evaluation of all of the properties, ACR LST (activated), which contains butadiene rubber, was selected for further research.

### 3.2. Object under Study: Crumb Rubber and Hydrocarbon Plasticizers

#### Determination of the Compatibility of Hydrocarbon Plasticizers with the Studied Crumb Rubber

Compatibility of hydrocarbon plasticizers with the studied crumb rubbers samples:

An important step in developing a stable and durable modified binder is the selection of hydrocarbon plasticizers compatible with the crumb rubbers polymers. Ensuring the compatibility of the component composition makes it possible to achieve the colloidal stability of the target product.

The use of plasticizers in the development of a modified binder naturally leads to the process of plasticization. According to the theory of the plasticization mechanism [50], it can occur through two mechanisms and at the following two structural levels:

1. Macromolecular: The plasticizer molecules are distributed among the macromolecules of the polymer.
2. Supramolecular: The plasticizer molecules are distributed among the supramolecular formations of the polymer.

Plasticization occurs via the first mechanism in cases of high thermodynamic affinity and via the second mechanism in cases of reduced surface tension during interaction. It is believed that molecular plasticization occurs through an energetic mechanism, while supramolecular plasticization occurs through a kinetic mechanism. Thus, based on the existing information [50], it can be assumed that the first plasticization mechanism is most preferable for obtaining a stable and durable binder modified with crumb rubbers. In this case, the plasticizer, when introduced into the polymer, “blocks” the polar groups, preventing them from interacting with each other, resulting in fewer nodes forming among the polymer’s macromolecules. This is also consistent with the “geometric effect” rule, which involves reducing spatial constraints during the movement of macromolecule segments.

To determine the possible plasticization mechanism, thermodynamic and chemical compatibility parameters were calculated using Formulas (1)–(12). These calculations were based on preliminary data on the group composition (Table 11) and surface tension of the hydrocarbon plasticizers (Table 12). The results of the compatibility parameters calculation are presented in Table 13.

An analysis of the obtained data (Table 13) indicates some differences in the presented compatibility criteria. It is generally accepted that thermodynamic parameters are more preferable because of their higher predictive capability. However, the following pattern was established: the values of the Hildebrand parameters (interaction parameters X1 and X2), calculated based on the evaporation energy criterion and surface tension, were similar only for the primary hydrocarbon plasticizers (residual extract and plasticizer RN-21). These plasticizers were characterized by the minimal standard deviation of the obtained values. In contrast, for the secondary plasticizers (waste industrial oil and purified waste frying oil), the standard deviation was more than twice as high. Additionally, according to Gibbs’ theory, the smaller the ∆δ = min, the more compatible the system components. However, for waste oils, there are divergent results in the solubility parameters calculated based on the different physical characteristics of these substances. For instance, by the evaporation energy parameter, secondary polymers can be classified as more compatible, while by the surface tension criterion, they are less compatible. Obviously, further research is required to determine the causes of these discrepancies. Therefore, considering the above, the chemical compatibility parameters, based on the criterion that the best compatibility is shown by plasticizers with a higher maltene content, which interacts better with polymers from crumb rubber, were used as selection criteria for the plasticizers. It is noteworthy that the Burshtein’s interaction parameter (|X|) for all studied hydrocarbon plasticizers (Table 13) exhibited similar values and, therefore, was not informative for the studied dispersed systems. However, it is important to note that the range of |X| values for all studied substances exceeded 0.55, which characterizes them as partially compatible or incompatible plasticizers.

Only one of the investigated hydrocarbon plasticizers, purified waste frying oil, meets both compatibility criteria. According to the data obtained (Table 13), this plasticizer had the highest Traxler dispersion coefficient and the mass ratio of paraffin-naphthenic compounds to asphaltenes was ∞ because of the absence of asphaltenes in the purified waste frying oil. Based on the analysis of the data presented in Table 13, purified waste frying oil was selected at this stage of the research for the development of crumb-rubber-modified binder.

### 3.3. Object under Study: Crumb-Rubber-Modified Binder

#### Determination of the Homogeneity, Solubility, and Delamination of the Obtained Crumb-Rubber-Modified Binder

In accordance with the selected experimental design plan, nine compositions of crumb-rubber-modified binder were prepared. The basic properties determining the composition’s effectiveness are the homogeneity of CR distribution and the solubility and degree of stratification, which characterize the binder’s stability. The solubility indicator is the most critical characteristic, as according to Russian legislation, without achieving this indicator’s required value, the crumb-rubber-modified binder cannot be used in road construction. During the research, it was found that all of the studied compositions did not meet the solubility requirement (Table 14).

An analysis of the presented results (Table 14) indicates that even the use of the activated crumb rubber ACR LST did not ensure the achievement of a stable and sustainable structure in crumb-rubber-modified binder. All CRMB compositions were heterogeneous and failed to meet the solubility and stratification criteria. Thus, the previously proposed hypothesis is confirmed—for the studied dispersed systems of “hydrocarbon plasticizer—crumb rubber”, the supramolecular plasticization mechanism was insufficient to produce stable, thermodynamically resilient crumb-rubber-modified binder.

Crumb rubber activation is usually related to partial surface devulcanization of the crumb rubber. This makes the crumb rubber surface more developed. This was previously thought to be sufficient for effective interaction with plasticizers and bitumen. However, our experiment demonstrated that in the systems under consideration, merely employing supramolecular plasticization is insufficient to achieve the required solubility criterion. At this stage of research, based on the research data obtained, we can formulate the following scientific hypothesis: the formation of a thermodynamically stable dispersed system of “bitumen—plasticizer—crumb rubber” should be based on the implementation of both the supramolecular mechanism (often applied in practice) and the molecular plasticization mechanism. This requires partial (controlled) physical devulcanization of the crumb rubber to reduce the energy barrier for subsequent processes of swelling, post-swelling, secondary destruction, and solubility. Therefore, the authors of this work propose expanding the existing understanding of the mechanism of effective interaction with crumb rubbers [10,11,12,13,14,15,16,17,18,19,20,21,22,23,24,25,26,27,28,29,30,31,32,33,34,35,36,37,38,39,40], as described in the analytical part of the study, by introducing the following additional first stage: preliminary controlled partial physical devulcanization of crumb rubbers.

The results of testing this hypothesis will be presented in the second part of the article, along with some structural parameters of the crumb-rubber-modified binder. Specifically, it will be shown that the particle size of the tire regenerate within the crumb-rubber-modified binder structure was less than 2 μm. It was established that the rheological parameters of the structure characterizing the intermolecular interaction—such as the shear stability G*/sin δ for the original and RTFOT-aged binders—ranged from 1.16 to 4.64 kPa (>1 kPa) at a temperature of 64 °C and from 2.29 to 8.27 kPa (>2.2 kPa) at a temperature of 64 °C, respectively. The elastic structural component of the crumb-rubber-modified binder (creep phase–recovery phase), determined by the parameter of relative irreversible deformation J_3,2_, did not exceed 2.6 kPa (<4.5 kPa) at a temperature of 64 °C. It is also shown that the rheological structural parameter for the fatigue resistance, characterizing the durability and reliability of road pavements under intensive usage and evaluated by cyclic bending loads simulating real-world conditions, did not exceed 4699 kPa (<5000 kPa) at 16 °C.

## 4. Conclusions

It was found that the average mass ratio of the polymer component in the investigation crumb rubber was 93.3 ± 1.8%. The stability of the polymer components in the investigation crumb rubber taken from different batches and sizes was analyzed using pyrolytic gas chromatography-mass spectrometry. It was established that they were characterized by identical polymers, leading to the conclusion that the raw materials and final crumb rubbers products produced at the same plant were stable.

It was found that the ACR LST sample had the lowest glass transition temperature at 9.59 °C, while the nonactivated NCR LST and CR CRP 0.5 samples had values 34% and 100% higher, respectively. The temperature at which destruction occurred for all samples studied was within the range of 300 to 310 °C. So, the activated CR LST sample is a promising modifier in terms of the temperature range for property retention. It was found that both nonactivated and activated crumb rubbers from large-sized tires were based on butadiene rubber, while CR CRP 0.5 was based on butadiene–styrene rubber. Considering that butadiene rubber has high elasticity over a wide temperature range and in accordance with the existing knowledge about the best interaction of activated crumb rubber with bitumen, activated crumb rubber was selected for further research.

An analysis of the compatibility parameters allowed for the identification of differences among them when using different characteristics. Stability in the calculated values was observed only for chemical compatibility parameters. Therefore, chemical compatibility parameters were used as criteria to justify the selection of compatible components. They were based on the condition of achieving better compatibility by those plasticizers characterized has having a large content of maltene, which is the most compatible with polymers in crumb rubbers. Only purified waste frying oil meets two compatibility chemical criteria at once.

It was found that for the studied dispersed systems of “hydrocarbon plasticizer–crumb rubber” the supramolecular plasticization mechanism was insufficient to produce sustainable crumb-rubber-modified binder. It was found that the use of activated-crumb-rubber ARC LST did not ensure the achievement of a stable and sustainable structure in crumb-rubber-modified binder. All compositions of crumb-rubber-modified binder were heterogeneous and did not meet the requirements for solubility and delamination.

A scientific hypothesis was formulated arguing that the formation of a thermodynamically stable dispersed system of “bitumen–plasticizer–crumb rubber” should be based on the realization of a supramolecular mechanism (often applied in practice) and a molecular plasticization mechanism. This requires the partial (controlled) physical devulcanization of crumb rubber to reduce the energy barrier of the subsequent swelling, postswelling, secondary destruction, and solubility. The results of testing this hypothesis are expected to be presented in a second article.

## Figures and Tables

**Table 1:** Physical properties of the studied plasticizers.

Indicator	Hydrocarbon Plasticizers, Actual Indicator			
	ROE	WIO	PWFO	RN-21
Kinematic viscosity at 50 °C, mm^2^/c	905.01	21.88	32.99	191.64
Kinematic viscosity at 100 °C, mm^2^/c	47.69	5.33	8.46	21.83
Flash point, °C	284.3	220.5	300	208

**Table 2:** Physical and mechanical properties of BND 70/100 bitumen.

Indicator	Requirements of Russian State Standard GOST 33133-14	Actual Indicator
Needle penetration depth, 0.1 mm, at a temperature of 25 °C	71–100	82
Needle penetration depth, 0.1 mm, at a temperature of 0 °C	more 21	26
Softening temperature of the ring and ball, °C	more 47	48.4
Ductility, cm, at 0 °C	more 3.7	4.1
Fragility, °C	less −18	−20
Change in the mass of the sample after aging, %	less 0.6	0.28
Change in the softening temperature of the sample after aging, %	less 7	6

**Table 3:** Selected planning parameters for the development of the modifier solution’s composition for the crumb-rubber-modified binder (CRMB).

Factor	Main Level, X_0_	Interval, ΔX
X_1_	9	1.0
X_2_	8.5	1.5

**Table 4:** Planning matrix for the development of the composition of CRMB.

Composition Number	In Encoded Values		In Natural Values	
	X_1_	X_2_	X_1_	X_2_
1	−1	−1	8	7
2	1	−1	10	7
3	−1	1	8	10
4	1	1	10	10
5	−1.41	0	7.59	8.5
6	1.41	0	10.41	8.5
7	0	−1.41	9	6.38
8	0	1.41	9	10.62
9	0	0	9	8.5

**Table 5:** Mass ratio of the polymer component (hydrocarbons) in the crumb rubbers.

Crumb Rubber	Mass Fraction of Rubber Hydrocarbons, %	Research Method
NCR LST	94.6	Russian State Standard GOST R 54550-2011 Synthetic rubber. Determination of the mass fraction of extracted substances
ACR LST	95.1	
CR CRP 0.5	91.5	
The average value and the range of variation	93.3 ± 1.8%	

**Table 6:** Polymers in the crumb rubbers under study.

No.	Exit Time, min	Decomposition Products				
		NCR LST		ACR LST		CR CRP 0.5
		fr. 0.8 mm	fr. 0.6 mm	fr. 0.8 mm	fr. 0.6 mm	fr. 0.5 mm
1	0.846	Isobutylene	Isobutylene	Butene-2	Butene-2	Butene-2
2	0.922	2-Methylbuta-diene 1,3	2-Methylbuta-diene 1,3	2-Methylbuta-diene 1,3	2-Methylbuta-diene 1,3	2-Methylbuta-diene 1,3
3	1.282	1-Hexen-3-yne	1-Hexen-3-yne	1,3,5-Cycloheptatriene	1,3,5-Cycloheptatriene	1-Hexen-3-yne
4	2.220	1,3,5-Cycloheptatriene	1,3,5-Cycloheptatriene	1,3,5-Cycloheptatriene	1,3,5-Cycloheptatriene	1,3,5-Cycloheptatriene
5	3.168	-	-	-	-	4-Ethenylcyclohexene
6	3.712	Xylene	Xylene	Xylene	Xylene	Xylene
7	4.025	-	-	-	-	Styrene
8	5.260	Isomer/homolog of limonene	Isomer/homolog of limonene	Isomer/homolog of limonene	Isomer/homolog of limonene	Isomer/homolog of limonene
9	6.546	Limonene	Limonene	Limonene	Limonene	Limonene
10	6.621	Diisobutylene	Diisobutylene	Diisobutylene	Diisobutylene	Diisobutylene

**Table 7:** Summary of the chemical elements and their contents on the surface of the crumb rubber samples.

Crumb Rubber	Elements and Their Surface Contents on the Studied Sample													
	C	O	Mg	Al	Si	S	K	Ca	Fe	Cu	Zn	Mn	Cl	Na
NCR LST	85.52	7.83	0.07	1.14	1.54	0.94	0.05	0.23	0.51	0.25	1.9	-	-	-
ACR LST	74.91	12.52	0.18	1.71	2.03	0.91	0.15	0.64	4.22	0.5	2.15	0.06	-	-
CR CRP 0.5	87.83	5.83	0.10	0.74	0.57	1.50	0.10	0.52	0.57	-	2.21	-	0.09	-

The method does not fix the element H and, as a rule, N.

**Table 8:** Thermodynamic parameters of the crumb rubbers samples under study.

Crumb Rubber	Temperature, °C, at
	Destruction
NCR LST	300–310
ACR LST	300–310
CR CRP 0.5	300–310

**Table 9:** Nature of the polymer component in the crumb rubbers.

Crumb Rubber	Nature of the Polymer in Crumb Rubber	Research Method
NCR LST	Butadiene rubber	Qualitative analysis of organic nonvolatile components by pyrolytic gas chromatography-mass spectrometry based on the products of their thermal decomposition (600 °C)
ACR LST	Butadiene rubber	
CR CRP 0.5	Styrene butadiene rubber	

**Table 10:** Comparative analysis of the properties of the elastomeric rubbers.

Butadiene Rubber (BR)	Styrene–Butadiene Rubber (SBR)
ρ = 900–920 kg/m^3^	ρ = 920–930 kg/m^3^
High elasticity over a wide temperature range	High elastic recovery
Ability to deform reversibly at low stresses	High Mooney viscosity
Resistance to low temperatures	High stiffness

**Table 11:** Results of determining the group hydrocarbon composition of plasticizers.

Content of Hydrocarbon Groups, % by Weight:	ROE	WIO	PWFO	RN-21
- Oils, including:	75.6	82.8	99.6	79.1
Paraffin-naphthenic	22.5	62.7	6.2	34.6
Aromatic, including:	53.1	20.1	93.4	44.5
light aromatics	14.6	12.9	1.1	8.5
medium aromatics	13.9	4.0	0.3	10.3
heavy aromatics	24.6	3.2	92.0	25.7
- Resins, including:	16.0	10.2	0.4	18.1
Resins I	5.4	3.2	0.4	6.4
Resins II	10.6	7.0	0.0	11.7
- Asphaltenes	8.4	7.0	0.0	2.8

**Table 12:** Results of measuring the surface tension of hydrocarbon plasticizers.

Hydrocarbon Plasticizer	Surface Tension, mN/m
Residual oil extract (ROE)	48.14
Waste industrial oil (WIO)	25.98
Purified waste frying oil (PWFO)	25.10
Plasticizer “RN-21”	34.41

**Table 13:** Compatibility parameters of hydrocarbon plasticizers with polymers from crumb rubbers.

Semi-Empirical Compatibility Parameters	ROE	WIO	PWFO	RN-21
Chemical parameters				
Traxler’s dispersibility coefficient (↑)	2.2	0.4	15.1	1.7
The mass ratio of paraffin-naphthenic compounds to asphaltenes (↑)	2.7	9.0	-	12.4
Burshtein’s interaction parameter (|X|)	0.97	0.99	0.98	0.98
Thermodynamic parameters				
Hildebrand compatibility parameters based on the evaporation energy criterion				
Flash point, K	557.5	493.7	573.2	481.2
Constant equal, J/(mole × K)	89.12	89.12	89.12	89.12
The energy of evaporation of organic substance, J/mole	49,679.9	43,994.1	51,079.1	42,880.1
Molar volume of the organic substance, cm^3^/mole	772.0	277.9	422.1	613.3
Solubility parameters of the plasticizer, δP1, (J/cm^3^)^0.5^	8.0	12.6	11.0	8.4
Solubility parameters of the modifier, δM1, (ACR LST), (J/cm^3^)^0.5^	29.3			
∆δ1	21.3	16.7	18.3	20.9
Solubility parameters—X1	94.3	21.2	38.3	72.6
The Hildebrand compatibility parameters based on the thermodynamic criterion—surface tension				
Surface tension, mN/m	48.137	25.979	25.103	34.414
Molecular weight, g/mole	747.9	240.6	388.7	585.0
Density at 20 °C, kg/m^3^ (Russian State Standard GOST 3900 [58])	968.8	865.8	920.8	953.8
Relative density ($\rho_{4}^{20}$)	0.969	0.866	0.921	0.954
Molar volume, cm^3^/mole	772.0	277.9	422.1	613.3
Solubility parameters of the plasticizer, δP2, (J/cm^3^)^0.5^	8.57	7.46	6.84	7.53
Solubility parameters of the modifier, δM2, (ACR LST), (J/cm^3^)^0.5^	29.3			
∆δ2	20.7	21.8	22.4	21.7
Solubility parameters—X2	89.5	35.9	57.5	78.5
Standard deviation for ∆δ1 and ∆δ2	0.39	3.62	2.94	0.59
Standard deviation for the solubility parameters X1 and X2	3.37	10.41	13.58	4.14

**Table 14:** Results of determining the properties characterizing the homogeneity and stability of the crumb-rubber-modified binder structures.

Composition Number	Homogeneity, Russian State Standard GOST R 52056	Solubility, % Russian State Standard GOST 33135-2014 (>99%)	Delamination (Russian State Standard GOST EN 13399-2013) and Differences in:	
			Softening Temperature, °C (˂3 °C)	Penetration Depth of a Needle at 25 °C, °C (˂20, 0.1 mm)
1	heterogeneous	does not correspond	6.1	26
2	heterogeneous	does not correspond	-	-
3	heterogeneous	does not correspond	-	-
4	heterogeneous	does not correspond	-	-
5	heterogeneous	does not correspond	6.3	21
6	heterogeneous	does not correspond	8.4	38
7	heterogeneous	does not correspond	-	
8	heterogeneous	does not correspond	-	-
9	heterogeneous	does not correspond	6.5	24

## Data Availability

Data are contained within the article.
